# The ANI-1ccx and ANI-1x data sets, coupled-cluster and density functional theory properties for molecules

**DOI:** 10.1038/s41597-020-0473-z

**Published:** 2020-05-01

**Authors:** Justin S. Smith, Roman Zubatyuk, Benjamin Nebgen, Nicholas Lubbers, Kipton Barros, Adrian E. Roitberg, Olexandr Isayev, Sergei Tretiak

**Affiliations:** 1 Center for Nonlinear Studies, Los Alamos National Laboratory, Los Alamos, NM USA; 2 Theoretical Division, Los Alamos National Laboratory, Los Alamos, NM USA; 3 Department of Chemistry, Carnegie Mellon University, Pittsburgh, PA USA; 4 Computer, Computational, and Statistical Sciences Division, Los Alamos National Laboratory, Los Alamos, NM USA; 5 University of Florida, Department of Chemistry, PO Box 117200, 32611-7200 Gainesville, USA

**Keywords:** Method development, Cheminformatics, Density functional theory

## Abstract

Maximum diversification of data is a central theme in building generalized and accurate machine learning (ML) models. In chemistry, ML has been used to develop models for predicting molecular properties, for example quantum mechanics (QM) calculated potential energy surfaces and atomic charge models. The ANI-1x and ANI-1ccx ML-based general-purpose potentials for organic molecules were developed through active learning; an automated data diversification process. Here, we describe the ANI-1x and ANI-1ccx data sets. To demonstrate data diversity, we visualize it with a dimensionality reduction scheme, and contrast against existing data sets. The ANI-1x data set contains multiple QM properties from 5 M density functional theory calculations, while the ANI-1ccx data set contains 500 k data points obtained with an accurate CCSD(T)/CBS extrapolation. Approximately 14 million CPU core-hours were expended to generate this data. Multiple QM calculated properties for the chemical elements C, H, N, and O are provided: energies, atomic forces, multipole moments, atomic charges, etc. We provide this data to the community to aid research and development of ML models for chemistry.

## Abstract (summary)

**Table:** 

Measurement(s)	Quantum Mechanics • energy • force • multipole moment • atomic charge
Technology Type(s)	computational modeling technique
Factor Type(s)	atom
Sample Characteristic - Environment	organic molecule

Machine-accessible metadata file describing the reported data: 10.6084/m9.figshare.12046440

## Background & Summary

Machine learning (ML) and data driven methods have far reaching applications across much of science and engineering. The power of machine learning stems from its ability to generalize predictions to unseen samples. Formulation of accurate and general-use ML models, especially neural network models, requires data sets that maximally span the problem space of interest. As real world examples of this need, data sets for autonomous vehicles require data from varying geographic locations, diverse weather conditions, urban and rural areas, while data sets for teaching autonomous drones to fly require crash scenarios to perform well^1^. A grand challenge for the machine learning community is determining the best way to develop sufficiently diverse data sets. Robotics has spearheaded the efforts to build such data sets through the use of active learning^2^: building data sets by asking ML models to choose what data needs to be added to a training set to perform better next time. Although the concept of active learning originates from robotics, it has recently grown into an extremely important tool for collecting quantum chemistry data sets for use in ML applications^3–11^.

Building an optimally diverse data set for training ML models is a problem-specific task. In this work, we define diversity in data as an adequate coverage of the problem space of interest that allows ML algorithms to generalize to unseen samples from many sources. In chemistry, diverse data sets can be difficult to come by. For example, ML has been applied for the prediction of chemical reactions based on experimental data, but models can be biased due to the lack of available data where reactions fail^12^. Such biases can be attributed to a lack of diversity in the training data. Herr *et al*. show standard molecular dynamics sampling is an inefficient method for sampling in the development of ML potentials because it leads to a lack of diversity in data^13^. An important task for any researcher applying ML is to determine what represents a sufficiently diverse data set to build general use models in their domain.

In chemistry, ML methods have been developed for the prediction of *ab initio* quantum mechanics (QM) computed properties, e.g. molecular potential energies and forces^14–18^, atomic charges^19–21^, molecular dipoles^22^, HOMO-LUMO gaps, and more^23,24^. ML has also been applied for the prediction of QM-based Hamiltonians^25,26^, which provide the electronic structure for a given system. Machine learning approaches promise to revolutionize the computational study of atomic systems by providing a highly accurate and computationally efficient route for obtaining QM accurate properties. These methods are typically thousands to millions of times faster than reference electronic structure methods, which use the most advanced numerical algorithms. ML methods for QM property prediction utilize various techniques including kernel ridge regression (KRR)^27,28^, Gaussian process regression (GPR)^29,30^, neural networks^15,18,31–34^ and others^35–37^.

Several benchmark data sets for measuring the prediction accuracy of ML-based QM property predictors have been publicly released. The QM9^23^ and QM7^24^ data sets have become staple benchmarks in the field. The QM7 benchmark consists of 7 k molecules with their associated energies while QM9 consists of multiple QM properties from up to 130 k different molecules computed with density functional theory (DFT). A subset of QM9 was also computed with highly accurate G4MP2 calculations. To date, many ML methods have achieved mean absolute errors (MAE) for molecular potential energy prediction on the order of 0.2 kcal/mol on the QM9 benchmark^16,18,38^, with 1 kcal/mol being considered the “gold standard” of chemical accuracy. These benchmarks have led to many successes in the field of QM property prediction, especially considering early models had MAE on the order of 5 to 10 kcal/mol on the QM7 benchmark^27^. The recently released Alchemy data set aims to extend the molecular size of data in the QM9 data set from 9 non-hydrogen atoms to 14^39^. The QM7, QM9 and similar benchmarks aim to train predictive models of QM properties, such as potential energies, at energy minimized molecular geometries computed using DFT. Since these data sets only span chemical space but the structures are at their equilibrium ground state geometries, ML models trained to them are only shown to provide accurate predictions for molecular structures obtained with an expensive DFT geometry optimization. Recent work by Lu *et al*. aims to alleviates this problem using force field optimized geometries and transfer learning^40^. Other benchmarks utilize single molecule MD trajectories^15^. The goal of single molecule benchmarks is to measure the accuracy of an ML model for learning the local potential surface of a given molecule. While both types of aforementioned benchmarks, equilibrium molecules and single molecule potential surface, have been pioneers in the field by providing a basis of comparison for different models, neither has aimed to cover both the chemical (connectivity of different elements) and conformational (same connectivity but differing geometries) spaces of molecules, simultaneously.

More recent research has focused on the construction of data sets with millions of highly diverse data points with the goal of creating a general-purpose ML-based potential^18,41,42^. The data for training such highly flexible ML models must contain the necessary information for predicting a complete potential energy surface for a class of molecules. Constructing these data sets represents a challenging case since the dimensionality of the problem is very high, and the conformational space of a molecule is not known *a priori*. The ANI-1 data set^43^, which consists of over 20 million conformations derived from 57 thousand distinct molecular configurations containing the C, H, N, and O chemical elements, is an example of such a data set. This data set was used to build the general-purpose ANI-1 potential, which was shown to accurately predict the non-equilibrium potential surface of molecules much larger than those included in the training data set. The ISO-17 benchmark^15^, another example of a benchmark that spans both chemical and conformational space, contains 5000 molecular conformations extracted from finite temperature MD simulations of 129 isomers with the chemical formula C_7_O_2_H_10_. Finally, the ChemSpider data set^42^ is comprised of potential energies and forces of 3 million conformations from 15 thousand different C, H, N, and O containing molecules from the ChemSpider database.

In this data descriptor, we provide the ANI-1x^5^ and ANI-1ccx^44^ data sets to the broader scientific community. The ANI-1x data set contains DFT calculations for approximately five million diverse molecular conformations. These conformations were obtained through an active learning algorithm, whereby the ANI ML potential is trained in an iterative manner to determine what new data should be included in future versions of the training data set. The fact that the ANI-1x and ANI-1ccx data sets are generated through non-equilibrium sampling rather than equilibrium sampling means that these data set must contain a more diverse set of conformations than existing QMx style data sets. The ANI-1x data set also contains vastly more diverse molecular conformations than those supplied in the original ANI-1 random sampled data set since active learning methods were employed in its construction. After many iterations of active learning, the resulting data set, dubbed the ANI-1x data set, was employed to train the ANI-1x potential. The ANI-1ccx data set is an intelligently selected 10% sub-sample of the ANI-1x data set, but recomputed with an accurate coupled cluster (approximately CCSD(T)/CBS) level of theory. Portions of the ANI-1x data set were recomputed with a larger basis set using the wb97x DFT functional for later work^31^. The data sets are provided in one easy to access HDF5 file along with scripts and examples needed to extract the data. The combination of all aforementioned DFT and coupled cluster variants of the ANI-1x data sets, with a selection of properties, are supplied to the scientific community in this data descriptor. Future extensions of these data sets will add new chemical elements and more molecular diversity will also be released to the community^45^.

## Methods

These methods are expanded versions of descriptions in our related work on developing general-purpose machine learning potentials for organic molecules through active learning^5^ and transfer learning^44^.

### Active learning data selection

**Fig. 1:**
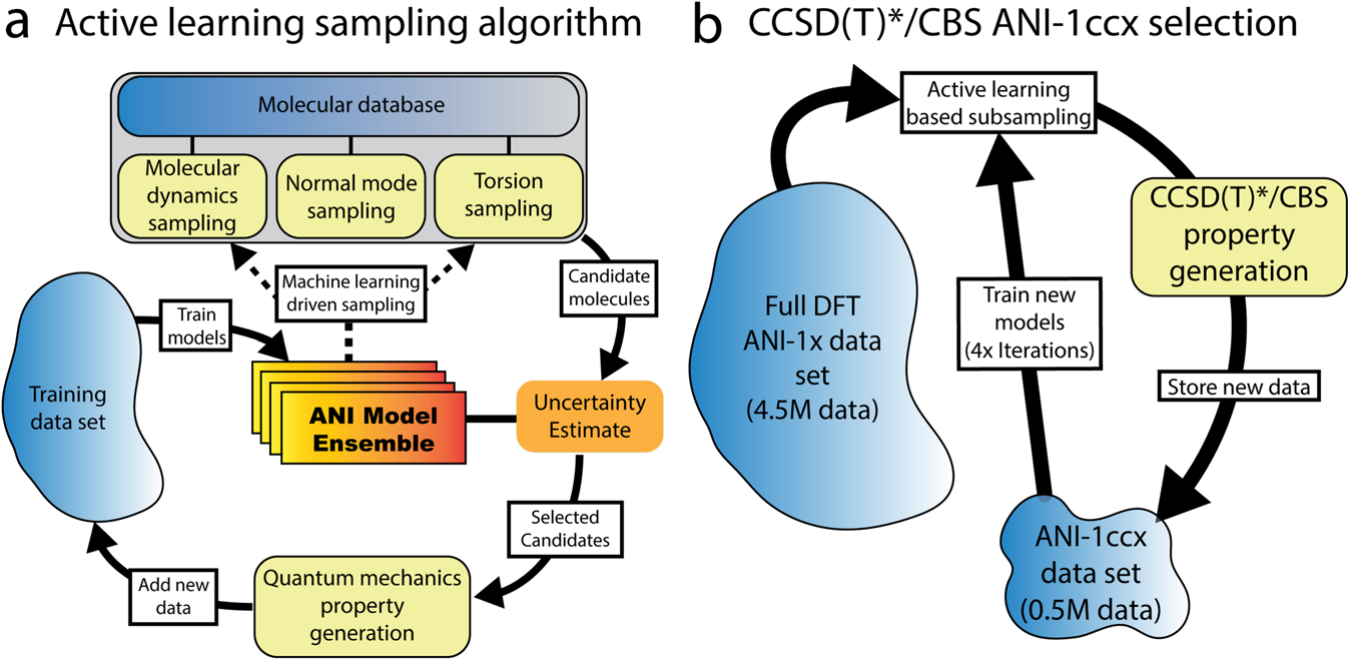
Active learning schemes for building ANI data sets. (**a**) The active learning algorithm employed during the construction of the ANI-1x data set. (**b**) The ANI-1ccx selection and data generation scheme.

The initial ANI-1x data set was generated as a part of an active learning procedure to develop the ANI-1x potential^5^. Active learning is where an ML model is used to determine what new data should be included in later generations to improve predictive ability. Figure 1a depicts the active learning algorithm. First, an ensemble of ANI models is trained to an initial bootstrap data set. Databases of molecules such as GDB-11^46,47^, ChEMBL^48^, and generated (with the RDKit cheminformatics python package^49^) amino acids and 2-amino acid peptides are randomly sampled for new molecule configurations, and one of four types of active learning sampling techniques is carried out on each of the selected molecules. These sampling techniques include molecular dynamics sampling, normal mode sampling, dimer sampling, and torsion sampling. These methods are described further in then Sampling methods section. Our active learning procedure involves a search through chemical and conformational space, employing a measure of estimated uncertainty to choose what new data should be generated, then including the new data in the next training cycle. The uncertainty estimate provides *a priori* information about the ensembles predictive performance. The uncertainty estimate employed in the ANI-1x active learning is based on an ensemble disagreement measure, henceforth referred to as *ρ*. The value *ρ* is proportional to the standard deviation of the prediction of an ensemble of ML models, normalized by the square root of the number of atoms in the system. It is described in detail in a previous publication^5^. When the uncertainty metric hints that a given molecular structure is poorly described (i.e., a large *ρ* value), new DFT data is generated and added to the training data set. The ensemble of models is then retrained with the new data added to the original data set. The new data is added in batches to accelerate the active learning process. The entire process is carried out iteratively to produce a successively more diverse data set, and hence a more robust ML model. In this work we use molecular dynamics (MD) simulations and geometry optimizations with our ML models. These simulations are performed with the Atomic Simulation Environment (ASE) python based library^50^.

### Sampling methods

#### Molecular dynamics sampling

The molecular dynamics (MD) sampling method utilizes the currently trained ML model ensemble to drive an MD simulation of a randomly selected molecule. The MD simulation is carried out at a random temperature between 50 K and 800 K using the Langevin thermostat, a time step of 0.5 fs, and a friction coefficient of 0.02 fs^−1^. Every 5 MD time steps *ρ* is computed for the molecular structure *x* at that time step. If the measure *ρ* is larger than a predetermined value, *x* is selected and added to a set $\bar{X}$ of high *ρ* molecules. The MD simulation is terminated once *x* is selected, because we assume the current potential no longer describes the atomic physics of the system at that point. DFT data is generated for all molecules in $\bar{X}$ and added to the training data set.

#### Normal mode sampling

This sampling technique^41^ begins with the QM generation of normal modes for tens of thousands of molecules. The molecules are selected from the GDB-11 and ChEMBL^48^ databases by employing the ensemble disagreement value *ρ*. When a molecule is selected the geometry is optimized to the local energy minima, then normal modes and harmonic force constants are calculated with QM and stored. During normal mode sampling the normal modes and harmonic force constants are used to generate a set of N conformations {*x*_*i*_}, where *i* indexes the conformation. The conformations are generated by displacing the atoms a random distance along each normal mode. All N conformations are then tested with the current ANI ensemble to obtain *ρ*_*i*_. All *ρ*_*i*_ larger than a predetermined threshold are selected then added to a set $\bar{X}$ of high *ρ* molecular conformations. DFT data is generated for all molecules in $\bar{X}$ and added to the training data set.

#### Dimer sampling

Dimer sampling starts with the random selection of a rectangular periodic cell size within the range of 20 to 30 angstroms. Molecules from a subset of the GDB-11 database are then selected randomly, with a higher probability of choosing molecules with less non-hydrogen atoms. The selected molecules are then embedded within the adopted periodic cell with random positions and rotation, ensuring that no two atoms in different molecules are within a distance of 1.5 Å. The atom density of the box is also randomly determined within a range. The current generation ANI potential is used to run an MD simulation on the constructed box of molecules. MD is carried out at a random temperature between 50 K and 600 K using the Langevin thermostat, a time step of 0.5 fs, and a friction coefficient of 0.02 ${{\rm{fs}}}^{-{\rm{1}}}$. After 100 time steps, the box is decomposed into a complete set of dimer structures {*x*_*i*_}, where *i* indexes the dimers. Only dimer structures with at least two atoms, one from each monomer, within a distance cutoff of 6 Å are selected. The ML model ensemble is then used to determine ${\rho }_{i}$ for each *i* dimer, and if the dimer has a ${\rho }_{i}$ value greater than a predetermined cutoff, the dimer is added to a set $\bar{X}$ of high $\rho$ dimers. DFT data is generated for all molecules in $\bar{X}$ and added to the training data set.

#### Torsion sampling

The torsion sampling active learning cycles are carried out after the original ANI-1x data set is produced as a part of building the ANI-1ccx potential^44^. A SMILES^51^ string is selected from a set of drug molecules containing relevant torsions^52^. RDKit is used to embed the molecules in 3D space and select a rotate-able bond, i.e. a torsion. The current generation ML potential is used to optimize the starting geometry, and carryout a relaxed scan, incremented by 10 degrees over the entire torsion profile. At each of the 36 scan steps, $\rho$ is measured. If $\rho$ is larger than a predetermined threshold the scan is halted and the structure *x* is stored. The scan is halted at the point where $\rho$ is high because we assume that the potential should not be trusted to continue performing the scan. Normal modes and harmonic force constants for *x* are generated using the current generation ANI potential ensemble. *x* is then randomly perturbed along those normal modes to generate four additional slightly perturbed conformations. This is done to sample the space around the valley of the torsion profile, rather than directly sampling the path through the valley. The latter could lead to a potential that poorly describes the overall shape of the reference QM potential. The four perturbed structures are added a set $\bar{X}$ of high $\rho$ structures. DFT data are generated for all molecules in $\bar{X}$ and added to the training data set. A total of 20 iterations of this torsional sampling protocol are carried out on all molecules in the Sellers *et al*. set^52^.

### High-throughput coupled cluster calculation scheme

ML potentials can archive errors lower than 1 kcal/mol compared to the underlying QM reference method^16,38,41^. As ML methods become more accurate at fitting to reference data, it becomes advantageous to develop data sets from more accurate levels of theory. An accurate and affordable QM method is the coupled cluster method with the inclusion of single, double and perturbative triple excitations, i.e., CCSD(T). This method is usually referred to as the “gold standard” of computational chemistry methods. Recently, an approximate DLPNO-CCSD(T)^53^ method was developed and implemented in the ORCA software package^54^. This method has a linear scaling with system size and is 10–100 times less expensive for medium-sized molecules (10–30 atoms). The error of the DLPNO approximation is controlled in the ORCA package with NormalPNO and TightPNO option presets. The TightPNO setting usually introduces a very small error, compared to the error of the pristine CCSD(T) method itself^55^. Besides the electronic level of theory, an extrapolation towards the complete basis set (CBS) limit is also a requirement to obtain high accuracy reference QM data. A very accurate and computationally efficient composite extrapolation scheme developed by Hobza and Sponer^56^ uses an MP2/(aug-)cc-pV[TQ]Z extrapolation and CCSD(T) correlation energy correction calculated with the (aug-)cc-pVTZ basis set. We used a similar idea to construct a composite extrapolation scheme, which is 50 times faster than full CCSD(T)/CBS for an aspirin-size molecule, without considerable sacrifice of accuracy^44^.

The components of our extrapolation scheme, dubbed CCSD(T)*/CBS, are the following: the effect of increasing the DLPNO approximation accuracy setting is estimated as the difference between TighPNO and NormalPNO CCSD(T) calculations with the cc-pVDZ basis set. The DLPNO-CCSD(T)/cc-pVTZ energy is calculated with a NormalPNO setting. The difference between cc-pVTZ and CBS correlation energy is estimated with an extrapolation of MP2 energy using the cc-pVTZ and cc-pVQZ basis sets. The HF/CBS energy is estimated with an extrapolation of cc-pVTZ and cc-pVQZ energies. The final equation for our CCSD(T)*/CBS composite extrapolation scheme is:

1 $$\begin{array}{lll}{{\rm{E}}}_{{\rm{CBS}}}^{{\rm{CCSD(T)}}\ast } & = & {E}_{{\rm{xtrap}}}({\rm{3/4,HF}})+\,{E}_{{\rm{xtrap}}}({\rm{3/4,MP2}})-\,E({\rm{3,MP2}})\\  &  & +\,E({\rm{3,NPNO}}-{\rm{CCSD(T)}})+\,E({\rm{2,TPNO}}-{\rm{CCSD(T)}})\\  &  & -\,E({\rm{2,NPNO}}-{\rm{CCSD(T)}})\end{array}$$

where E(N, Method) refers to the energy of ‘Method’ calculated with basis set of cardinal number N (2, 3 and 4 for cc-pVDZ, cc-pVTZ, and cc-pVQZ), NPNO and TPNO are Normal and Tight DLPNO respectively, and Extrap(N1/N2, Method) refers to the complete basis set extrapolation performed with formulas from Halkier^57^ and Helgaker^58^:

2 $${{\rm{E}}}_{{\rm{xtrap}}}(3/4,{\rm{HF}})=\frac{{e}^{-\alpha \sqrt{4}}{E}_{HF}^{(3)}-{e}^{-\alpha \sqrt{3}}{E}_{HF}^{(4)}}{{e}^{-\alpha \sqrt{4}}-{e}^{-\alpha \sqrt{3}}}$$

3 $${{\rm{E}}}_{{\rm{xtrap}}}(3/4,{\rm{corr}})=\frac{{4}^{\beta }{E}_{corr}^{(4)}-{3}^{\beta }{E}_{corr}^{(3)}}{{4}^{\beta }-{3}^{\beta }}$$

Parameters *α* = 5.46 and *β* = 3.05 optimized for cc-pV[TQ]Z extrapolation are used from a previous report^59^.

### CCSD(T)*/CBS data selection

Since our CCSD(T)*/CBS extrapolation scheme is significantly more computationally expensive than the original ANI-1x DFT reference calculations, only a subset of the original data could be computed using the extrapolation scheme. Therefore, we used a subset sampling technique similar to the original active learning method to select what data to compute at the CCSD(T)*/CBS level of theory. Figure 1b depicts the subset selection technique; first, a uniformly random subset of the ANI-1x data set is selected, then CCSD(T)*/CBS reference data is generated. An ensemble of models is trained to this new CCSD(T)*/CBS data set. The ensemble disagreement value $\rho$ is generated for all remaining ANI-1x data points. A subset of the data with $\rho$ greater than a predetermined value is selected, and CCSD(T)*/CBS reference data is generated. This data is added to the CCSD(T)*/CBS training data set. The cycle was carried out four times to generate the final ANI-1ccx data set. Supplemental information Tables 1 and 2 provides counts for all data in the ANI-1ccx data set.

## Data Records

All data records [10.6084/m9.figshare.c.4712477] are provided in a single HDF5^60^ file which is hosted by figshare^61^. The provided HDF5 file can be iterated through using a python script named “example_loader.py”, which we provide in a publicly accessible GitHub repository [https://github.com/aiqm/ANI1x_datasets]. Using our extraction scripts and examples, all data can easily be accessed. The Usage Notes section provides more details about how to access the data from the provided HDF5 file. The property name along with the dictionary keys, units, data type, and NumPy array shape, are provided in Table 1. Tables S1 and S2 in the supplemental information contains counts for all data in this article. Three primary electronic structure methods were used to generate the data in this article: wB97x/6-31G*, wB97x/def2-TZVPP, and CCSD(T)*/CBS. Many secondary methods for the CCSD(T)*/CBS extrapolation are also included. All wB97x/6-31G* calculations were carried out in the Gaussian 09^62^ electronic structure package, while all wB97x/def2-TZVPP and CCSD(T)*/CBS calculations were performed in the ORCA^54^ software package. Table S3 contains a map between the key value and the level of theory at which the property was computed. In all, we include multiple types of energies, forces, electronic multipole moments, and charges obtained from the Hirshfeld and CM5 charge partitioning schemes. Minimal basis iterative stockholder partitioning (MBIS) scheme^63^ implemented in the HORTON software library^64^ was used to calculate atomic charges, volumes, and the magnitude of higher order atomic multipoles up to octupoles based on the wB97x/def2-TZVPP electron density.

**Table 1:** Data layout in the provided HDF5 file. Nc is the number of conformations and Na is the number of atoms.

Property	Key	Units	Type	Shape
Atomic Positions	‘coordinates’	Å	float32	(Nc, Na, 3)
Atomic Numbers	‘atomic_numbers’	—	uint8	(Na)
Total Energy	‘wb97x_dz.energy’	Ha	float64	(Nc)
	‘wb97x_tz.energy’			
	‘ccsd(t)_cbs.energy’			
HF Energy	‘hf_dz.energy’	Ha	float64	(Nc)
	‘hf_tz.energy’			
	‘hf_qz.energy’			
NPNO-CCSD(T)	‘npno_ccsd(t)_dz.corr_energy’	Ha	float64	(Nc)
Correlation	‘npno_ccsd(t)_tz.corr_energy’			
Energy	‘npno_ccsd(t)_qz.corr_energy’			
MP2	‘mp2_dz.corr_energy’	Ha	float64	(Nc)
Correlation	‘mp2_tz.corr_energy’			
Energy	‘mp2_qz.corr_energy’			
Atomic Forces	‘wb97x_dz.forces’	Ha/Å	float32	(Nc, Na, 3)
	‘wb97x_tz.forces’			
Molecular	‘wb97x_dz.dipole’	e Å	float32	(Nc, 3)
Electric	‘wb97x_tz.dipole’			
Moments	‘wb97x_tz.quadrupole’	e *AA*^2^		(Nc, 6)
Atomic	‘wb97x_dz.cm5_charges’	e	float32	(Nc, Na)
Charges	‘wb97x_dz.hirshfeld_charges’			
	‘wb97x_tz.mbis_charges’			
Atomic	‘wb97x_tz.mbis_dipoles’	a.u.	float32	(Nc, Na)
Electric	‘wb97x_tz.mbis_quadrupoles’			
Moments	‘wb97x_tz.mbis_octupoles’			
Atomic Volumes	‘wb97x_tz.mbis_volumes’	a.u.	float32	(Nc, Na)

## Technical Validation

### Diversity comparison

Here, we compare the diversity of atomic environments sampled in ANI-1x and ANI-1ccx to two prior data sets: QM9 and ANI-1. The QM9 data set^23^ is a collection of 134 k molecules with up to 9 non-hydrogen atoms (C, N, O, and F) along with molecular properties (electronic and vibrational energies, Highest Occupied Molecular Orbitals (HOMO) and Lowest Unoccupied Molecular Orbital (LUMO) energies, dipole moments, etc.) calculated at the DFT optimized conformer geometry with the B3LYP/6-31G(2df,p) method. These molecules correspond to a subset of the GDB-17^65^ chemical universe of 166 billion organic molecules. The QM9 data set represented a molecular property set of unprecedented size and consistency at the time of its inception. Subsequently, this data set has become very popular for training ML property predictors for organic molecules^66–75^. However, the principal disadvantage of the QM9 data set is that it is composed of only optimized molecular structures. ML models trained on the QM9 data set should not be used for non-equilibrium simulations, such as MD. Further, applications of an ML model trained to QM9 requires a DFT geometry optimization to achieve accurate predictions, since all geometries in the training set were DFT optimized geometries.

The ANI-1 data set^43^ is the predecessor to the data sets presented in this work. The ANI-1 data set contains approximately 20 million randomly selected structures and and their corresponding DFT computed energies. Similar to the QM7 and QM9 data sets, the ANI-1 data set covers chemical degrees of freedom. However, most importantly, it also aims to describe conformational degrees of freedom by providing non-equilibrium molecular geometries for many different molecules. The nearly 20 million non-equilibrium geometries were generated by randomly deforming molecules along DFT computed vibrational normal modes. This process was carried out for approximately 50 k different molecules with up to 8 heavy (C, N, O) atoms. Training an ANI model to this data set resulted in the ANI-1 potential, a model capable of extending its predictive accuracy to molecules much larger than those in the training data set. However, the ANI-1 model was random sampled from an enumeration of small organic molecules from GDB-11^46,47^, which left the data set clustered, yet sparse, in conformational space coverage. The ANI-1x and ANI-1ccx data sets were generated to provide a more diverse sampling of conformational space for small organic molecules.

**Fig. 2:**
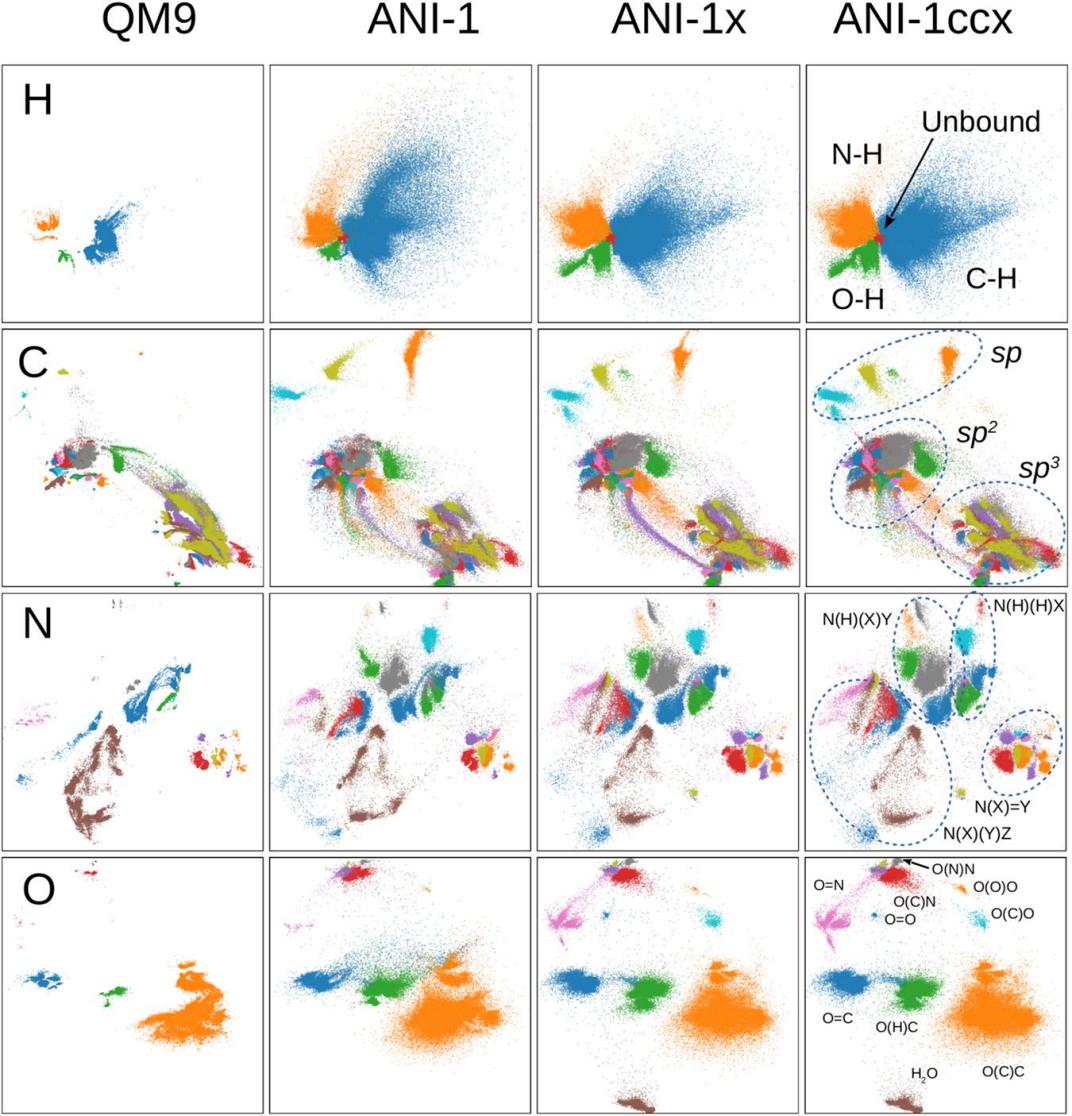
2D parametric t-SNE embeddings. These embeddings are for the 1st layer of activations of the ANI-1x model for the complete QM9 data set and random subsets of the ANI-1, ANI-1x and ANI-1ccx data sets. The same number of atoms are compared for each element. The different colors correspond to the number and type of bonded neighbors.

We use features of the environment of each atom to compare the chemical and conformational space coverage for the QM9, ANI-1, ANI-1x and ANI-1ccx data sets. Instead of comparing the atomic environment vectors (AEVs) of the ANI^41^ model, we use intermediate neural network activations of a trained model to compare the similarity of two chemical environments; specifically, we use the activation values that form the first layer of the ANI-1x^5^ model. We use activation values rather than the descriptors (AEVs) since AEVs tend to be sparse, making it difficult to use simple L2 distance between vectors as a similarity metric. The first layer extracts the most useful information from the AEV and reduces the dimensionality of the atomic descriptor from 384 to 160 (H), 144 (C) and 128 (N and O). For visual comparison, we applied the parametric t-Distributed Stochastic Neighbor Embedding (pt-SNE) technique^76^ which learns a neural network-based mapping from high-dimensional vectors to a 2-dimensional representation, while preserving both global and local structure of the data in high-dimensional space as much as possible. We used the complete ANI-1x data set to learn a pt-SNE mapping from the ANI-1x first layer activations to a 2D space using a deep neural network. Figure 2 provides a direct comparison of 2D embedding of atom environment features for the entire QM9 data set and random subsets of the ANI-1, ANI-1x, and ANI-1ccx data sets with the same number of atoms for each element. The 2D embeddings were learned from the ANI-1x data set. The colors correspond to the type and number of bonded neighbors as determined by the Openbabel software library^77^, e.g. the ethanol molecule has two types of C atoms with environment HHHC and HHCO. We also provide an interactive version on our supporting GitHub repository [https://github.com/aiqm/ANI1x_datasets], with all labels corresponding to the colors used.

Visual comparison of the pt-SNE embeddings clearly show atoms cluster according to the type and the local chemical environment. Carbon, for example, has distinct clusters corresponding to *sp*^3^, *sp*^2^ and *sp* hybridization for both QM9 and ANI data sets. All four data sets have a similar number of clusters, reflecting the chemical diversity of the data sets. The ANI-1x and ANI-1ccx data sets have noticeably more diffuse clusters compared to the QM9 data set. This fact reflects greater coverage of conformational space. Comparing the random conformational sampling of the ANI-1 data set with the active learning generated ANI-1x data sets shows a visually similar trend. However, upon close inspection it is clear that the ANI-1x data has a more diverse coverage of chemical space, especially for N and O. The coverage of the ANI-1x and ANI-1ccx data sets is very similar, which is expected since the ANI-1ccx data set is a sub-sampling of the ANI-1x data set.

### Energy and composition coverage

**Fig. 3:**
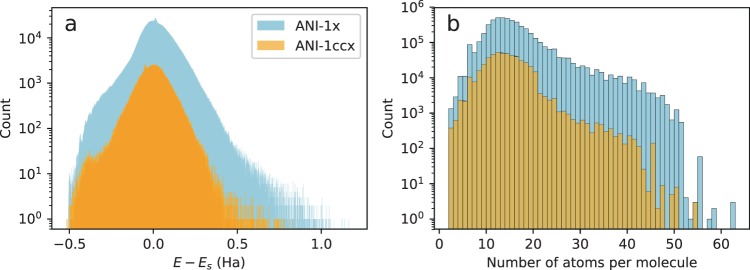
ANI data set energy and size distribution. (**a**) A histogram of the potential energies in the ANI-1x and ANI-1ccx data sets with a linear fit per atomic element *E*_*s*_ removed. The bin width is 1 millihartree. (**b**) A histogram of the total number of atoms (including C, H, N, and O atoms) per molecule in the ANI-1x and ANI-1ccx data sets. The bin width is one.

Figure 3a provides a histogram showing the distribution of energies in the data sets. The energies have had a constant shift removed, which is the sum of a linear fitting to the atomic elements. The figure also shows the energy range of applicability for models trained to this data set. The ANI-1ccx data set has a similar distribution as the ANI-1x data set since ANI-1ccx is an active learning-based sub-sample of ANI-1x. Figure 3b is a histogram of the number of atoms (C, H, N, and O) per molecule in each data set. After making note of the logarithmic scale on the y axis, it is clear that the vast majority of data in these data sets is derived from molecules with less than 20 atoms in total. The ANI-1ccx data set tends to have smaller molecules than the ANI-1x data set because the coupled cluster calculations on larger molecules sometimes fail to complete due to computer memory limitations.

## Usage Notes

We provide python based tools for extracting these data sets, along with code examples, on a public supporting GitHub repository [https://github.com/aiqm/ANI1x_datasets]. Box 1 provides an example of a python script for loading the ANI-1x wb97x/6-31G* data set from the provided ANI-1x HDF5 file. After importing the data loader in line 0, a function called ‘iter_data_buckets’ provides an iterator that can be used in a for loop (line 5) to access dictionaries containing all data for chemical isomers. Two arguments are passed to this function. The first argument is the path to the ani-1x HDF5 file (defined in line 2). The second is a list of keys describing what data will be loaded (defined in line 4). The script will only load conformers that share the requested data for property keys given in the ‘data_keys’ list. For example, if the ‘data_keys’ list contains two keys ‘wb97x_dz.energy’ and ‘ccsd(t)_cbs.energy’, then only conformers that share both energies will be loaded, approximately 500k structures. However, if one removes ‘ccsd(t)_cbs.energy’ from the list, then approximately 5 million structures will be loaded. Finally, the data (geometry coordinates, atomic numbers, and requested properties) stored in NumPy arrays can be accessed by requesting the specific key, as shown in lines 6–9. Note that the keys match those given in Table 1.

Box 1: Example use of the data loader script 0) import dataloader as dl

1)

2) path_to_h5file = ‘PATH/TO/H5/FILE/ani1x.h5’

3)

4) data_keys = [‘wb97x_dz.energy’,‘wb97x_dz.forces’]

5) for data in dl.iter_data_buckets(path_to_h5file,keys=data_keys):

6) X = data[‘coordinates’

7) Z = data[‘atomic_numbers’]

8) E = data[‘wb97x_dz.energy’]

9) F = data[‘wb97x_dz.forces’]

## Supplementary information

supplemental information

## Data Availability

All electronic structure calculations were computed with the Gaussian 09 [cite] or ORCA electronic structure packages [cite]. All molecular dynamics simulations for sampling were carried out with the atomic simulation environment (ASE). The analysis and active learning scripts are available upon request. The C++/CUDA implementation of our ANI code is available online in binary format [ref], but source code is not publicly released. Alternatively a PyTorch version ANI is available as open source. [https://github.com/aiqm/torchani].

## References

[1] Gandhi, D., Pinto, L. & Gupta, A. Learning to fly by crashing. In *2017 IEEE/RSJ International Conference on Intelligent Robots and Systems (IROS)*, 3948–3955 (IEEE, 2017).

[2] Settles B (2012). Active learning. Synthesis Lectures on Artificial Intelligence and Machine Learning.

[3] Reker, D. & Schneider, G. *Active-learning strategies in computer-assisted drug discovery*, vol. 20 (Elsevier Current Trends, 2015). 10.1016/j.drudis.2014.12.004 25499665

[4] Podryabinkin EV, Shapeev AV (2017). Active learning of linearly parametrized interatomic potentials. Computational Materials Science.

[5] Smith JS, Nebgen B, Lubbers N, Isayev O, Roitberg AE (2018). Less is more: sampling chemical space with active learning. The Journal of Chemical Physics.

[6] Gubaev K, Podryabinkin EV, Shapeev AV (2018). Machine learning of molecular properties: Locality and active learning. Journal of Chemical Physics.

[7] Gubaev K, Podryabinkin EV, Hart GL, Shapeev AV (2019). Accelerating high-throughput searches for new alloys with active learning of interatomic potentials. Computational Materials Science.

[8] Zhang L, Lin DY, Wang H, Car R, Weinan E (2019). Active learning of uniformly accurate interatomic potentials for materials simulation. Physical Review Materials.

[9] Bernstein, N., Csányi, G. & Deringer, V.L. De novo exploration and self-guided learning of potential-energy surfaces. *npj Comput Mater* **5**, 99 (2019).

[10] Deringer VL, Pickard CJ, Csányi G (2018). Data-Driven Learning of Total and Local Energies in Elemental Boron. Physical Review Letters.

[11] Nguyen TT (2018). Comparison of permutationally invariant polynomials, neural networks, and Gaussian approximation potentials in representing water interactions through many-body expansions. Journal of Chemical Physics.

[12] Raccuglia P (2016). Machine-learning-assisted materials discovery using failed experiments. Nature.

[13] Herr JE, Yao K, McIntyre R, Toth D, Parkhill J (2018). Metadynamics for Training Neural Network Model Chemistries: a Competitive Assessment. The Journal of Chemical Physics.

[14] Anderson, B., Hy, T.-S. & Kondor, R. Cormorant: Covariant Molecular Neural Networks. *arXiv* Preprint at: http://arxiv.org/abs/1906.04015 (2019).

[15] Schütt KT, Arbabzadah F, Chmiela S, Müller KR, Tkatchenko A (2017). Quantum-Chemical Insights from Deep Tensor Neural Networks. Nature Communications.

[16] Schütt KT, Sauceda HE, Kindermans PJ, Tkatchenko A, Müller KR (2018). SchNet - A deep learning architecture for molecules and materials. Journal of Chemical Physics.

[17] Suwa H (2019). Machine learning for molecular dynamics with strongly correlated electrons. Physical Review B.

[18] Unke, O. T. & Meuwly, M. PhysNet: A Neural Network for Predicting Energies, Forces, Dipole Moments, and Partial Charges. *Journal of Chemical Theory and Computation* **15**, 3678–3693 (2019). 10.1021/acs.jctc.9b00181 31042390

[19] Morawietz T, Sharma V, Behler J (2012). A neural network potential-energy surface for the water dimer based on environment-dependent atomic energies and charges. The Journal of Chemical Physics.

[20] Bleiziffer P, Schaller K, Riniker S (2018). Machine Learning of Partial Charges Derived from High-Quality Quantum-Mechanical Calculations. Journal of Chemical Information and Modeling.

[21] Nebgen, B. *et al*. Transferable Dynamic Molecular Charge Assignment Using Deep Neural Networks. *J. Chem. Theory Comput*., 10.1021/acs.jctc.8b00524 (2018). 10.1021/acs.jctc.8b00524 30064217

[22] Sifain AE (2018). Discovering a Transferable Charge Assignment Model Using Machine Learning. The Journal of Physical Chemistry Letters.

[23] Ramakrishnan, R., Dral, P. O., Rupp, M. & von Lilienfeld, O. A. Quantum chemistry structures and properties of 134 kilo molecules. *Scientific data* **1**, 140022, http://www.nature.com/articles/sdata201422 (2014). 10.1038/sdata.2014.22 PMC4322582 25977779

[24] Montavon G (2013). Machine learning of molecular electronic properties in chemical compound space. New Journal of Physics.

[25] Li H, Collins C, Tanha M, Gordon GJ, Yaron DJ (2018). A Density Functional Tight Binding Layer for Deep Learning of Chemical Hamiltonians. Journal of Chemical Theory and Computation.

[26] Welborn M, Cheng L, Miller TF (2018). Transferability in Machine Learning for Electronic Structure via the Molecular Orbital Basis. Journal of Chemical Theory and Computation.

[27] Rupp M, Tkatchenko A, Muller K-R, von Lilienfeld OA (2012). Fast and accurate modeling of molecular atomization energies with machine learning. Physical review letters.

[28] Collins CR, Gordon GJ, von Lilienfeld OA, Yaron DJ (2018). Constant size descriptors for accurate machine learning models of molecular properties. The Journal of Chemical Physics.

[29] Bartók AP, Payne MC, Kondor R, Csányi G (2010). Gaussian Approximation Potentials: The Accuracy of Quantum Mechanics, without the Electrons. Physical Review Letters.

[30] Fujikake S (2018). Gaussian approximation potential modeling of lithium intercalation in carbon nanostructures. Journal of Chemical Physics.

[31] Zubatyuk R, Smith JS, Leszczynski J, Isayev O (2019). Accurate and transferable multitask prediction of chemical properties with an atoms-in-molecules neural network. Science Advances.

[32] Yao K, Herr JE, Brown SN, Parkhill J (2017). Intrinsic Bond Energies from a Bonds-in-Molecules Neural Network. Journal of Physical Chemistry Letters.

[33] Lee K, Yoo D, Jeong W, Han S (2019). SIMPLE-NN: An efficient package for training and executing neural-network interatomic potentials. Computer Physics Communications.

[34] Herr JE, Koh K, Yao K, Parkhill J (2019). Compressing physics with an autoencoder: Creating an atomic species representation to improve machine learning models in the chemical sciences. The Journal of Chemical Physics.

[35] Thompson AP, Swiler LP, Trott CR, Foiles SM, Tucker GJ (2015). Spectral neighbor analysis method for automated generation of quantum-accurate interatomic potentials. Journal of Computational Physics.

[36] Ferré G, Haut T, Barros K (2017). Learning molecular energies using localized graph kernels. Journal of Chemical Physics.

[37] Bartók AP, Kondor R, Csányi G (2013). On representing chemical environments. Physical Review B - Condensed Matter and Materials Physics.

[38] Lubbers N, Smith JS, Barros K (2018). Hierarchical modeling of molecular energies using a deep neural network. The Journal of Chemical Physics.

[39] Chen, G. *et al*. Alchemy: A Quantum Chemistry Dataset for Benchmarking AI Models. *arXiv* Preprint at: https://arxiv.org/abs/1906.09427 (2019).

[40] Lu, J., Wang, C. & Zhang, Y. Predicting Molecular Energy Using Force-Field Optimized Geometries and Atomic Vector Representations Learned from an Improved Deep Tensor Neural Network. *Journal of Chemical Theory and Computation* 4113–4121 (2019). 10.1021/acs.jctc.9b00001 PMC6615995 31142110

[41] Smith J, Isayev O, Roitberg A (2017). ANI-1: an extensible neural network potential with DFT accuracy at force field computational cost. Chemical Science.

[42] Yao K, Herr JE, Toth DW, Mcintyre R, Parkhill J (2017). The TensorMol-0.1 Model Chemistry: a Neural Network Augmented with Long-Range Physics. Chemical Science.

[43] Smith JS, Isayev O, Roitberg AE (2017). Data Descriptor: ANI-1, A data set of 20 million calculated off-equilibrium conformations for organic molecules. Scientific Data.

[44] Smith JS (2019). Approaching coupled cluster accuracy with a general-purpose neural network potential through transfer learning. Nature Communications.

[45] Devereux, C. *et al*. Extending the Applicability of the ANI Deep Learning Molecular Potential to Sulfur and Halogens. *ChemRxiv* Preprint, 10.26434/chemrxiv.11819268.v1 (2020). 10.1021/acs.jctc.0c00121 32543858

[46] Fink T, Raymond JL (2007). Virtual exploration of the chemical universe up to 11 atoms of C, N, O, F: Assembly of 26.4 million structures (110.9 million stereoisomers) and analysis for new ring systems, stereochemistry, physicochemical properties, compound classes, and drug discove. Journal of Chemical Information and Modeling.

[47] Fink T, Bruggesser H, Reymond JL (2005). Virtual exploration of the small-molecule chemical universe below 160 daltons. Angewandte Chemie - International Edition.

[48] Davies M (2014). MyChEMBL: A Virtual Platform for Distributing Cheminformatics Tools and Open. Data. Challenges.

[49] Landrum, G. RDkit: Open-source Cheminformatics, http://www.rdkit.org.

[50] Hjorth Larsen A (2017). The atomic simulation environment - A Python library for working with atoms. Journal of Physics Condensed Matter.

[51] SMILES strings, www.opensmiles.org.

[52] Sellers BD, James NC, Gobbi A (2017). A Comparison of Quantum and Molecular Mechanical Methods to Estimate Strain Energy in Druglike Fragments. Journal of Chemical Information and Modeling.

[53] Guo Y (2018). Communication: An improved linear scaling perturbative triples correction for the domain based local pair-natural orbital based singles and doubles coupled cluster method [DLPNO-CCSD(T)]. The Journal of Chemical Physics.

[54] Neese F (2012). The ORCA program system. Wiley Interdisciplinary Reviews: Computational Molecular Science.

[55] Paulechka E, Kazakov A (2017). Efficient DLPNO-CCSD(T)-Based Estimation of Formation Enthalpies for C-, H-, O-, and N-Containing Closed-Shell Compounds Validated Against Critically Evaluated Experimental Data. The Journal of Physical Chemistry A.

[56] Hobza P, Šponer J (2002). Toward true DNA base-stacking energies: MP2, CCSD(T), and complete basis set calculations. Journal of the American Chemical Society.

[57] Halkier A, Helgaker T, Jørgensen P, Klopper W, Olsen J (1999). Basis-set convergence of the energy in molecular Hartree-Fock calculations. Chemical Physics Letters.

[58] Helgaker T, Klopper W, Koch H, Noga J (1997). Basis-set convergence of correlated calculations on water. The Journal of Chemical Physics.

[59] Neese F, Valeev EF (2011). Revisiting the Atomic Natural Orbital Approach for Basis Sets: Robust Systematic Basis Sets for Explicitly Correlated and Conventional Correlated ab initio Methods?. Journal of Chemical Theory and Computation.

[60] The HDF Group. Hierarchical Data Format, version 5, http://www.hdfgroup.org/HDF5 (2016).

[61] Smith JS (2020). figshare.

[62] M. J. Frisch, G. *et al*. Gaussian 09, Revision E.01 (2009).

[63] Verstraelen T (2016). Minimal Basis Iterative Stockholder: Atoms in Molecules for Force-Field Development. Journal of Chemical Theory and Computation.

[64] Verstraelen, T. *et al*. HORTON 2.1.0 (2017).

[65] Ruddigkeit L, Van Deursen R, Blum LC, Reymond JL (2012). Enumeration of 166 billion organic small molecules in the chemical universe database GDB-17. Journal of Chemical Information and Modeling.

[66] Faber FA, Christensen AS, Huang B, Von Lilienfeld OA (2018). Alchemical and structural distribution based representation for universal quantum machine learning. Journal of Chemical Physics.

[67] Eickenberg M, Exarchakis G, Hirn M, Mallat S, Thiry L (2018). Solid harmonic wavelet scattering for predictions of molecule properties. Journal of Chemical Physics.

[68] Gómez-Bombarelli R (2018). Automatic Chemical Design Using a Data-Driven Continuous Representation of Molecules. ACS Central Science.

[69] Chen C, Ye W, Zuo Y, Zheng C, Ong SP (2019). Graph Networks as a Universal Machine Learning Framework for Molecules and Crystals. Chemistry of Materials.

[70] Faber, F. A. *et al*. Prediction errors of molecular machine learning models lower than hybrid DFT error. *Journal of Chemical Theory and Computation* acs.jctc.7b00577 (2017). 10.1021/acs.jctc.7b00577 28926232

[71] Grattarola D, Livi L, Alippi C (2019). Adversarial autoencoders with constant-curvature latent manifolds. Applied Soft Computing Journal.

[72] Nikolentzos, G. & Vazirgiannis, M. Message Passing Graph Kernels. *arXiv* preprint arXiv:1808.02510, http://arxiv.org/abs/1808.02510 (2018).

[73] Kearnes, S., Li, L. & Riley, P. Decoding Molecular Graph Embeddings with Reinforcement Learning. *arXiv* preprint arXiv:1904.08915, http://arxiv.org/abs/1904.08915 (2019).

[74] Sinitskiy, A. V. & Pande, V. S. Deep Neural Network Computes Electron Densities and Energies of a Large Set of Organic Molecules Faster than Density Functional Theory (DFT). *arXiv* Preprint arXiv:1809.02723, http://arxiv.org/abs/1809.02723 (2018).

[75] von Rudorff, G. F. Molecular shape as a (useful) bias in chemistry. *arXiv* Preprint arXiv:1904.07035, http://arxiv.org/abs/1904.07035 (2019).

[76] van der Maaten, L. Learning a Parametric Embedding by Preserving Local Structure. In van Dyk, D. & Welling, M. (eds.) *Proceedings of the Twelth International Conference on Artificial Intelligence and Statistics*, vol. 5 of *Proceedings of Machine Learning Research*, 384–391 http://proceedings.mlr.press/v5/maaten09a.html (PMLR, Hilton Clearwater Beach Resort, Clearwater Beach, Florida USA, 2009).

[77] O’Boyle, N. M. *et al*. Open Babel: An Open chemical toolbox. *Journal of Cheminformatics* (2011). 10.1186/1758-2946-3-33 PMC3198950 21982300

[78] Sfiligoi, I. *et al*. The pilot way to Grid resources using glideinWMS. In *2009 WRI World Congress on Computer Science and Information Engineering, CSIE 2009*, vol. 2, 428–432 (IEEE, 2009).

[79] Pordes, R. *et al*. The open science grid. In *Journal of Physics: Conference Series*, vol. 78, 012057 (IOP Publishing, 2007).

