# Supplementary material for: The ANI-1ccx and ANI-1x data sets, coupled-cluster and density functional theory properties for molecules
Source: Sci Data. 2020 May 1;7:134. doi: 10.1038/s41597-020-0473-z (PMC7195467; doi:10.1038/s41597-020-0473-z)
Supplement: Supplementary file 1 — supplemental information [file 41597_2020_473_MOESM1_ESM.pdf]

Supplemental information for: *"The ANI-1ccx and ANI-1x data sets, coupled-cluster and density functional theory properties for molecules"*

Justin S. Smith[1,2,⋄], Roman Zubatyuk[2,5,⋄], Benjamin Nebgen[2], Nicholas Lubbers[3], Kipton Barros[2], Adrian E. Roitberg[*4], Olexandr Isayev[†5] and Sergei Tretiak[‡2]

[1]Center for Nonlinear Studies, Los Alamos National Laboratory, Los Alamos, NM
[2]Theoretical Division, Los Alamos National Laboratory, Los Alamos, NM
[3]Computer, Computational, and Statistical Sciences Division, Los Alamos National Laboratory, Los Alamos, NM
[4]University of Florida, Department of Chemistry, PO Box 117200, Gainesville, USA 32611-7200
[5]University of North Carolina at Chapel Hill, Division of Chemical Biology and Medicinal Chemistry, UNC Eshelman School of Pharmacy, Chapel Hill, USA 27599

List of Tables

1 Property Index used in supplemental information Table 2. 2
2 Counts of data with matching geometries. 2
3 Map from data set key to QM method/basis set. 3

*roitberg@ufl.edu
†olexandr@olexandrisayev.com
‡serg@lanl.gov
⋄ equal contribution to this work

Table 1: Property Index used in supplemental information Table 2.

Key	Property ID
wb97x_dz.energy	P1
wb97x_dz.forces	P2
wb97x_dz.dipole	P3
wb97x_dz.quadrupole	P4
wb97x_dz.hirshfeld_charges	P5
wb97x_tz.energy	P6
wb97x_tz.mbis_charges	P7
ccsd(t)_cbs.energy	P8

Table 2: Counts of data with matching geometries.

	P1	P2	P3	P4	P5	P6	P7	P8
P1	4956005	4956005	2857397	2528080	4752778	4617229	4617229	489571
P2	4956005	4956005	2857397	2528080	4752778	4617229	4617229	489571
P3	2857397	2857397	2857397	2528080	2857367	2721818	2721818	296669
P4	2528080	2528080	2528080	2528080	2528050	2528049	2528049	265823
P5	4752778	4752778	2857367	2528050	4752778	4617229	4617229	470894
P6	4617229	4617229	2721818	2528049	4617229	4617229	4617229	459464
P7	4617229	4617229	2721818	2528049	4617229	4617229	4617229	459464
P8	489571	489571	296669	265823	470894	459464	459464	489571

Table 3: Map from data set key to QM method/basis set.

Key	QM Method/Basis set
wb97x_dz.energy	wB97x/6-31G*
wb97x_tz.energy	wB97x/def2-TZVPP
ccsd(t)_cbs.energy	CCSD(T)*/CBS
wb97x_dz.forces	wB97x/6-31G*
wb97x_tz.forces	wB97x/def2-TZVPP
hf_dz.energy	HF/cc-pVDZ
hf_tz.energy	HF/cc-pVTZ
hf_qz.energy	HF/cc-pVQZ
npno_ccsd(t)_dz.corr_energy	NPNO-CCSD(T)/cc-pVDZ
npno_ccsd(t)_tz.corr_energy	NPNO-CCSD(T)/cc-pVTZ
tpno_ccsd(t)_dz.corr_energy	TPNO-CCSD(T)/cc-pVDZ
mp2_dz.corr_energy	MP2/cc-pVDZ
mp2_tz.corr_energy	MP2/cc-pVTZ
mp2_qz.corr_energy	MP2/cc-pVQZ
wb97x_dz.dipole	wB97x/6-31G*
wb97x_tz.dipole	wB97x/def2-TZVPP
wb97x_tz.quadrupole	wB97x/def2-TZVPP
wb97x_dz.cm5_charges	wB97x/6-31G*
wb97x_dz.hirshfeld_charges	wB97x/6-31G*
wb97x_tz.mbis_charges	wB97x/def2-TZVPP
wb97x_tz.mbis_dipoles	wB97x/def2-TZVPP
wb97x_tz.mbis_quadrupoles	wB97x/def2-TZVPP
wb97x_tz.mbis_octupoles	wB97x/def2-TZVPP
wb97x_tz.mbis_volumes	wB97x/def2-TZVPP
